# Integration of the proteome and transcriptome reveals multiple levels of gene regulation in the rice *dl2* mutant

**DOI:** 10.3389/fpls.2015.00351

**Published:** 2015-06-17

**Authors:** Xiaoyan Peng, Zhongliang Qin, Guopeng Zhang, Yaomin Guo, Junli Huang

**Affiliations:** Key Laboratory of Biorheological Science and Technology, Ministry of Education, Bioengineering College, Chongqing UniversityChongqing, China

**Keywords:** rice (*Oryza sativa*), *drooping leaf*, midrib, proteome, transcriptome, correlation

## Abstract

Leaf vascular system differentiation and venation patterns play a key role in transporting nutrients and maintaining the plant shape, which is an important agronomic trait for improving photosynthetic efficiency. However, there is little knowledge about the regulation of leaf vascular specification and development. Here we utilized the rice midribless mutant (*dl2*) to investigate the molecular changes in transcriptome and proteome profiles during leaf vascular specification and differentiation. Using isobaric tags for relative and absolute quantification (iTRAQ) with digital gene expression (DGE) techniques, a nearly complete catalog of expressed protein and mRNA was acquired. From the catalog, we reliably identified 3172 proteins and 9,865,230 tags mapped to genes, and subsets of 141 proteins and 98 mRNAs, which were differentially expressed between the *dl2* mutant and wild type. The correlation analysis between the abundance of differentially expressed mRNA and DEPs (differentially expressed proteins) revealed numerous discordant changes in mRNA/protein pairs and only a modest correlation was observed, indicative of divergent regulation of transcription and translational processes. The DEPs were analyzed for their involvement in biological processes and metabolic pathways. Up- or down- regulation of some key proteins confirmed that the physiological process of vascular differentiation is an active process. These key proteins included those not previously reported to be associated with vascular differentiation processes, and included proteins that are involved in the spliceosome pathway. Together, our results show that the developmental and physiological process of the leaf vascular system is a thoroughly regulated and complicated process and this work has identified potential targets for genetic modification that could be used to regulate the development of the leaf vasculature.

## Introduction

The plant vascular system performs important functions, such as delivery of nutrients to the various plant tissues, supply of mechanical support, as well as acting as an effective long-distance communication system relating to abiotic and biotic conditions above and below ground (Lucas et al., 2013). Plant leaves are the principal organs for photosynthesis. Independent origins of the plant leaf in different plant families indicate that it is a necessary evolutionary adaptation for land plants to capture solar energy efficiently (Campitelli and Stinchcombe, 2013). Plant leaf vascular differentiation and venation patterns play a key role in determining plant shape, which is an important agronomic trait. Leaf vascular systems consist of a continuous network of interconnected cells throughout the leaf. Considerable effort has been made examining genetic control of vascular differentiation using vascular system mutants, with several factors identified that affect developmental and physiological processes of the plant vascular system (Mahonen et al., 2000; Dettmer et al., 2009; Ohashi-Ito and Fukuda, 2010). However, a global view of gene activity around the process of vascular differentiation is required. In spite of extensive descriptions about vascular development and patterning, the molecular and physiological mechanisms governing the strictly regulated process remain to be elucidated.

Deciphering the regulation networks of functional genes that coordinately control biological processes will reveal a more comprehensive description of the biological processes and will provide candidates for improving the fitness and performance of plants. Bottom-up profiling of transcripts or proteins is a powerful approach to analyze changes in biological processes such as development or response to environmental perturbations, which are also an essential aspect of systems biology. High-throughput tag-sequencing for digital gene expression (DGE) analysis is a new and powerful tool that permits deep coverage of transcriptome profiles and enables the examination of expression changes with a high dynamic range (Bentley, 2006). This approach supplies a more qualitative and quantitative detection of gene expression than previous microarray-based assays (‘t Hoen et al., 2008). Due to the low cost, transcriptional profiling is the main choice for many laboratories investigating plant development or adaptation to environmental stresses. However, since biological processes are ultimately controlled by proteins, interrogation of changes in the protein profile is crucial for providing an accurate picture of the events triggered. Also, due to post-translational turnover and alternate translation efficiency, mRNA abundance is not a reliable embodiment of protein abundance, and only a modest correlation of the two has been reported (Ideker et al., 2001; Chen et al., 2002; Rogers et al., 2008; Kuss et al., 2011). The technique of isobaric tags for relative and absolute quantification (iTRAQ) combined with LC-MS/MS is popular because of its high resolution and detailed protein expression profiles (Ross et al., 2004). Bioinformatic knowledge has made it possible to analyze and display the data more quickly and accurately (Guo et al., 2008). iTRAQ-based proteomics has been widely applied in systems ranging from pathogen induced stress response in plants to mammalian organ development (Palmieri et al., 2012; Moczulska et al., 2014). A recent study addressing the adjustment of mRNA and protein abundance in mammalian cells emphasized the importance of translational regulation of protein abundance (Schwanhaeusser et al., 2011), underlining the significance of a comprehensive view on gene expression.

This study investigates genome-wide expression of the proteome and transcriptome of plant leaf vascular differentiation in the rice midribless *dl2* mutant. In an attempt to have a global view of gene activity in the process of leaf vascular differentiation in rice, and probe candidate genes involved in plant vascular differentiation, we examined the abundance of transcripts and proteins by DGE and iTRAQ technology, respectively, and correlated the abundance difference between mRNAs and proteins. The result indicated that the transcriptomic and proteomic expression profiles were poorly correlated, and there is a modest correlation between transcript and protein levels. In addition, according to their functional annotation, we found some differentially expressed proteins (DEPs) which might be involved in leaf vascular patterning and may help to elucidate the *drooping leaf* phenotype in *dl2* mutants. Our analysis also revealed different control mechanisms that dictate gene activity, uncovering several novel aspects in the metabolism of leaf vascular differentiation in rice. This study provides new clues to address the relationship between the abundance of mRNAs and proteins in plant vascular patterning.

## Materials and methods

### Plant materials and growth conditions

The *dl2* mutant (MT) of rice (Huang et al., 2011) was isolated from indica cultivar “N45-2” (WT), and the corresponding WT was used as a control in all studies. Rice plants were cultivated in a greenhouse under a 12 h photoperiod (300 μmol photons m^−2^ s^−1^) and at 30°C. Humidity was controlled at approximately 60%. Both MT and WT were of the same age and grown under the same conditions. The leaves were sampled for analysis from two-month-old rice plants when the leaves were fully expanded and the MT plants displayed a *drooping leaf* phenotype. Two biological replicates for each experiment were performed for transcriptome and proteome analysis, and each with pooled leaves from 10 independent plants per replicate. In addition, the leaf samples were flash-frozen in liquid nitrogen and preserved at −80°C for subsequent quantitative real-time PCR (qPCR) analysis.

### Protein extraction

Protein extraction was performed based on a previous report (Yang et al., 2012), with minor modification. Briefly, leaf tissue was ground in liquid nitrogen, and suspended in the Lysis buffer (7 M Urea, 4% CHAPS, 2 M Thiourea, 40 mM Tris-HCl, pH 8.5, 1 mM PMSF, 2 mM EDTA). The proteins were reduced with DTT (10 mM, final concentration) at 56°C for 1 h and then alkylated by 55 mM IAM (final concentration) in the darkroom for 1 h. The reduced and alkylated protein mixtures were precipitated by adding 4 volumes of chilled acetone at −20°C overnight. After centrifugation at 4°C, 30,000 g, the pellet was dissolved in 0.5 M TEAB (Applied Biosystems, Milan, Italy) and sonicated on ice. After centrifuging at 30,000 g at 4°C, the supernatant was analyzed for protein concentration using the Protein Assay Kit (Bio-Rad, Hercules, CA, USA) based on the Bradford method using a BSA standard. The quality of each protein sample was evaluated by SDS-PAGE and samples that showed no degradation and good resolution with low background were kept at −80°C for further analysis.

### Protein iTRAQ labeling and SCX fractionation of peptides

Protein iTRAQ labeling was carried out by Huada Genomics Co., Ltd. (Shenzhen, China). The trypsin digestion protocol has been previously described in detail (Lan et al., 2011). Desalted peptides were labeled with iTRAQ reagents (Applied Biosystems, Foster City, CA, USA) according to the manufacturer's instructions. Two WT samples were labeled with reagent 116 and 117, and two MT samples were labeled with reagent 119 and 121, respectively. The reactions were controlled to proceed for 2 h at room temperature. Subsequently, the WT and the MT peptides were combined in equal parts and further fractionated offline using strong cation exchange (SCX) chromatography (LC-20AB, Shimadzu, Japan).

SCX chromatography was performed on a high performance liquid chromatography system (Shimadzu, Kyoto, Japan) using a 250 × 4.6 mm (5-μm particle size) ultremex SCX column (Shimadzu, Kyoto, Japan), based on the following study (Fan et al., 2011). A 10-min elution was used by buffer A (a combination of 25 mM NH_2_PO_4_ in 25% acetonitrile, pH 2.7), and followed by 11-min gradient with 5–35% buffer B (25 mM NH_2_PO_4_, 1 M KCl in 25% acetonitrile, pH 2.7), and then by ramping up to 80% solvent B for 1 min. The absorbance at 214 nm was monitored and a total of 20 fractions were collected, lyophilized and desalted using the Strata X column (Shimadzu, Kyoto, Japan). Eluents were lyophilized and stored at −80°C before LC-ESI-MS/MS analysis.

### LC-ESI-MS/MS mass spectrometry based on TripleTOF 5600

Liquid chromatography was performed using a LC-20AD nanoHPLC (Shimadzu, Kyoto, Japan) and loaded by an autosampler onto a 2 cm C18 trap column. Then, peptides were eluted onto a 10 cm analytical C18 column (inner diameter 75 μm) packed in-house. The samples were loaded at 8 μl/min for 4 min, then the 35 min gradient was run at 300 nL/min starting from 2 to 35% B (95% ACN, 0.1% FA), followed by 5 min linear gradient to 60%, then, followed by 2 min linear gradient to 80%, and maintenance at 80% B for 4 min, and finally return to 5% in 1 min.

Data acquisition was performed with a TripleTOF 5600 System (AB SCIEX, Concord, ON) fitted with a Nanospray III source (AB SCIEX, Concord, ON) and a pulled quartz tip as the emitter (New Objectives, Woburn, MA), based on a recent report (Wang et al., 2013; Guo et al., 2014). Data was acquired using an ion spray voltage of 2.5 kV, curtain gas of 30 psi, nebulizer gas of 15 psi, and an interface heater temperature of 150°C. The MS was performed with an RP of greater than or equal to 30,000 FWHM for TOF MS scans. For IDA, survey scans were acquired in 250 ms and as many as 30 product ion scans were collected if exceeding a threshold of 120 counts per second and with a 2+ to 5+ charge-state. Total cycle time was fixed to 3.3 s. Q2 transmission window was 100 Dafor 100%. Four-time bins were summed for each scan at a pulser frequency value of 11 kHz through monitoring of the 40 GHz multichannel TDC detector with four-anode channel detect ion. A sweeping collision energy setting of 35 ± 5 eV coupled with iTRAQ adjust rolling collision energy was applied to all precursor ions for collision-induced dissociation. Dynamic exclusion was set for 1/2 of peak width (15 s), and then the precursor was refreshed off the exclusion list.

### iTRAQ data analysis

Raw data files obtained from the TripleTOF 5600 System (AB SCIEX, Concord, ON) were transformed into MGF files using Proteome Discoverer 1.2 (Thermo Fisher Scientific), and the MGF file was searched. Protein identification was performed by using Mascot search engine (version 2.3.02) against a database containing 66,338 rice protein sequences (http://www.ricedata.cn/gene/).

For protein identification, a mass tolerance of ± 0.05 Da was permitted for intact peptide masses and ± 0.1 Da for fragment ions, with allowance for one missed cleavage by trypsin. Gln->pyro-Glu (N-term Q), Oxidation (M), Deamidated (NQ) as the potential variable modifications, and Carbamidomethyl (C), iTRAQ 8 plex (N-term), iTRAQ 8 plex (K) as fixed modifications. The charge states of peptides were set to +2 and +5. Specifically, an automatic decoy database search was carried out in Mascot by choosing the decoy checkbox. To reduce the probability of false peptide characterization, only peptides with scores (≥20) at 99% or greater confidence by Mascot probability analysis were considered as greater than “identity” and counted as identified, and each confident protein identification involved at least one unique peptide. For protein quantification, it was required that a protein contain at least two unique peptides. The quantitative protein ratios were evaluated and normalized by the median ratio in Mascot. We only used ratios with *p*-values < 0.05, and only a fold change ≥1.6 was considered as significant.

### RNA extraction, DGE library preparation and sequencing

Total RNA was obtained with the RNeasy Plant Mini Kit (Qiagen, Valencia, CA), and contaminating DNA was digested with the DNA-free Kit (Ambion, Austin, TX), according to the kit instructions. RNA quantity was measured in a NanoDrop ND-1000 spectrophotometer (NanoDrop Technologies, Wilmington, DE). RNA quality was evaluated with an Agilent Bioanalyzer Model 2100 (Agilent Technologies, Palo Alto, CA). mRNA was enriched by using oligo (dT) magnetic beads.

DGE library construction and sequencing were carried out at Huada Genomics Co., Ltd., (Shenzhen, China). Briefly, the mRNA was broken into short fragments (about 200 bp) by adding the fragmentation buffer, and then the cDNA was synthesized and single nucleotide A (adenine) was added, which is used for ligation of sequencing adaptors. Finally, the fragments were enriched by PCR amplification, and the enriched library products were used for sequencing analysis via Illumina HiSeq™ 2000.

### DGE tags analysis and gene expression annotation

DGE tag mapping followed previously-described protocols (Xue et al., 2010). The raw sequence files were filtered to clean tags, which were mapped to the gene tags (http://rice.plantbiology.msu.edu/). The number of explicit clean tags for each gene was computed and then normalized to TPM (number of transcripts per million clean tags, ‘t Hoen et al., 2008; Morrissy et al., 2009).

### Screening of differentially expressed genes (DEGs)

A novel approach employing Noiseq was used to characterize DEGs between WT and MT (Audic and Claverie, 1997; Tarazona et al., 2012). *P*-value corresponds to the test of differential gene expression. Here two biological replicates were performed; thus false discovery rate (FDR) was substituted with the *Q*-value to determine the threshold of *P*-value in multiple tests and analysis (Tarazona et al., 2012). We use both the *Q*-value ≥ 0.8 and absolute value of log_2_ratio ≥ 1 as the threshold to judge the significance of gene expression difference at *P* < 0.05 (Benjamini and Yekutieli, 2001).

### Quantitative real-time PCR (qPCR) validation

Quantitative real-time PCR (qPCR) analysis was employed to verify the DGE results using the thermocycler CFX1000 (Bio-Rad, CA) with SYBR Premix Ex Taq TMII (Perfect Real Time) kit (Takara). Primers for specific genes were designed using Primer Premier 5.0. qPCR assays were performed in triplicate (technical repeats) with two independent biological replicates, with β-*actin* as the internal control. The quantitative verification was performed by a relative quantitative method (2^−ΔΔCT^) (Pfaffl, 2001).

## Results

### Protein identification and quantification using iTRAQ

To investigate the proteome expression differences between WT and MT, proteins from two biological replicates per phenotype were analyzed with iTRAQ technology. Protein identities were acquired by searching the database containing rice protein sequences (http://www.ricedata.cn/gene/). Results were tested for the significance of protein identification and protein abundance variation using the Mascot search engine (Matrix Science, UK; version 2.3.02). A total of 8835 unique peptides and 3172 proteins from the two experiments were characterized with higher than 99% confidence as shown in supplementary data (Table S1) using the criteria described in the experimental procedures.

### DEPs revealed by iTRAQ in *dl2* mutant

**Figure 1:**
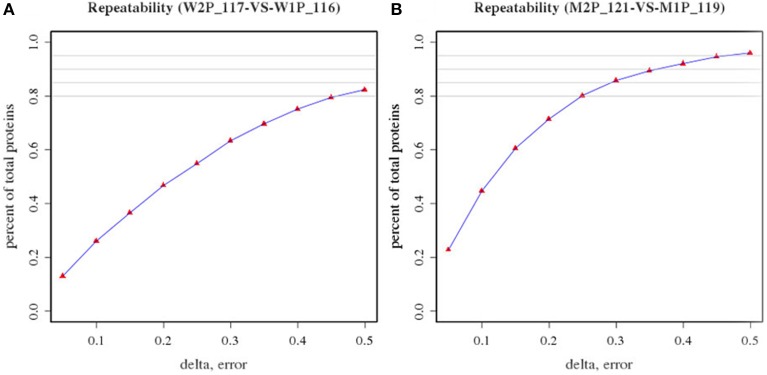
**Repeatability of two biological replicates of wild-type samples (tags 116 and 117) and *dl2* mutant samples (tags 121 and 119). (A)** Repeatability of two biological replicates of wild-type samples (W2P117 and W1P116); **(B)** Repeatability of two biological replicates of *dl2* mutant samples (M2P 121 and M1P119).

In an attempt to determine DEPs between WT and MT leaf samples, the repeatability of two biological replicates of WT (tags 116 and 117) and MT (tags 121 and 119) was evaluated first (Figure 1). The percentage of the total proteins between the two biological replicates in the two experiments was approximately 0.8 or more, thereby indicating the biological reproducibility of protein expression. Further, the biological variations between two replicates were estimated to set an optimal cut-off value of the fold change for determining significantly altered differential protein expression levels. Fold changes were generated between two biological replicates of control samples and MT samples, respectively. The variations within two biological replicates are presented in Table 1. Around 95% of proteins had fold changes less than 1.6 within biological replicates. Therefore, a 1.6-fold change was used as the optimal threshold to achieve 95% confidence (*P* < 0.05) for measuring DEPs between MT and WT samples. In addition, only proteins which were identified by two or more peptides and had ≥1.6-fold change of the ratios (tags 117/116 and 121/119) were considered to be DEPs.

**Table 1:** **Evaluation of biological variance of proteome data**.

**Percentage of proteins**	**Fold change**	
	**(117/116)**	**(121/119)**
99	1.933	1.676
95	1.583	1.384
90	1.375	1.247

*95% proteins had fold changes less than 1.6 within biological replicates*.

**Figure 2:**
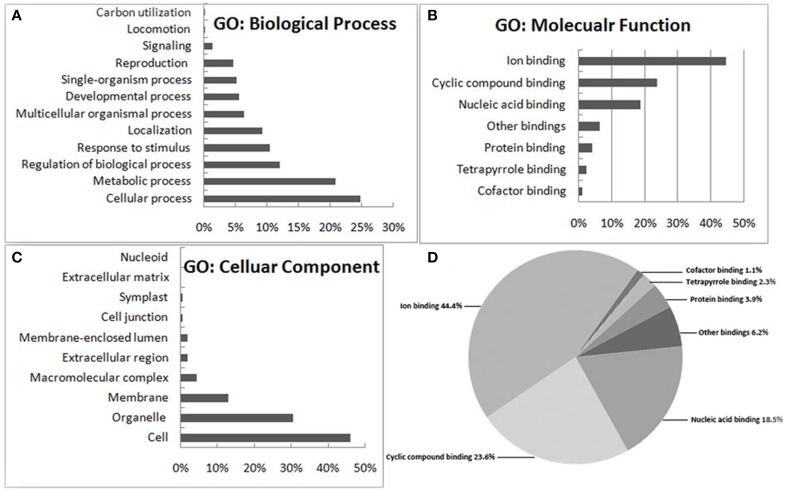
**Functional categorization of the differentially expressed proteins between wild type and *dl2* mutant samples**. The genes were categorized based on Gene Ontology (GO) annotation and the proportion of each category is displayed based on **(A)** biological process, **(B)** molecular function, or **(C)** cellular component. Specialized terms of the binding category **(D)** shows 141 sequences implicated in binding.

A total of 141 proteins showed a significant difference (*P* < 0.05) after the comparison (Table S2), of which 94 and 47 showed increased or decreased accumulation levels in MT compared to WT samples, respectively. DEPs were categorized according to their biological process, molecular function and cellular component using blast2GO program (http://www.blast2go.org/), respectively. A protein might be associated with or located in one or more cellular components, suggesting that it performs functions in one or more biological process. Among these, following the functional classification with the three unrelated GO ontologies, 82 (58%) are involved in biological processes, 82 (58%) have molecular functions, and 106 (75%) are cellular components (Figure 2). For each of these ontologies, annotated sequences are primarily distributed among two or more of the general term categories. Within the 82 differentially expressed proteins involved in biological processes, the main categories represented were cellular process (24.7%), metabolic process (20.8%), regulation of biological process (11.9%) and stress response (10.5%), respectively (Figure 2A). Similarly, in the molecular functions sub-ontology, 43.2% and 41.8% of the DEPs have catalytic activity and binding, respectively (Figure 2B). Finally, in the 106 unique proteins predicted to be cellular components, 46.2, 30.6, and 13.2% are related to cell, organelle and membrane components, respectively (Figure 2C). In the binding section of the category of molecular function, sequence distributions between specialized terms showed that the greatest numbers fell under ion-binding (44.4%) and cyclic compound binding (23.6%), with the third largest group from nucleic acid binding (18.5%) (Figure 2D). This result indicated that many of the DEPs are cellular components and involved in a wide variety of biological processes including development, metabolism and signal transduction. Of specific interest were proteins from the GO category of biological processes related to leaf vascular pattern formation and shoot system developmental processes.

### DEPs related to *drooping leaf* mutation revealed by iTRAQ analysis in *dl2* mutant

In order to analyze the DEPs related to the *drooping leaf* mutation in the *dl2* mutant, we listed the DEPs with change ratios ≥1.6-fold, which might be involved in biological processes related to leaf vascular formation and patterning (Table 2). These DEPs showed increased accumulation levels, such as SART-1 (LOC_Os02g30730.1) and RNA-binding protein (LOC_Os08g02390.1), or decreased accumulation levels, like lycerophosphoryl diester phosphodiesterase and peroxidases (LOC_Os04g39610.1). The SART-1 protein (LOC_Os02g30730.1) levels were increased significantly (ratio = 1.733) in the *dl2* mutant (Table 2). In Arabidopsis, leucine zipper protein SART-1 (human squamous cell carcinoma-associated reactive antigen for cytotoxic T-cells) encoded by *DOT2* is listed as a component of the spliceosome (Arabidopsis nucleolar protein database; http://bioinf.scri.sari.ac.uk/cgi-bin/atnopdb/home) and involved in cell cycle inhibition, apoptosis as a transcription factor (Gupta et al., 2000; Hosokawa et al., 2005; Petricka et al., 2008). The spurred-leaf class-*dot2* mutant from Arabidopsis is dwarfed, and the cotyledon number and vascular pattern are disturbed (Petricka et al., 2008). It has been reported that the RNA-binding protein U2AF (U2 small nuclear ribonucleoprotein auxiliary factor) is an essential splicing factor with critical roles in recognition of the splice site (Jenkins et al., 2013; Paulo Tavanez et al., 2013). Therefore, the RNA-binding protein (LOC_Os08g02390.1), acting as a component of splicesome, might play key roles in rice leaf vascular formation. It is known a variety of studies showed that the plant peroxidase is responsible for lignin biosynthesis during plant vascular development (Bargaz et al., 2013; Zhao et al., 2013; Shigeto et al., 2014). In summary, the results demonstrate that these DEPs might be involved in the physiological process of leaf vascular differentiation, but are not necessarily specific to this process.

**Table 2:** **A selection of proteins found to be related to *dl2* mutation**.

**ID**	**Description**	**Ratio (MT/WT)**	**Go annotation**	**Pathway**
LOC_Os02g30730.1	SART-1 family protein	+1.733	Leaf vascular tissue pattern formation/xylem and phloem pattern formation/ pattern specification process	Spliceosome
LOC_Os08g02390.1	RNA recognition motif containing protein	+1.652	Shoot system development/shoot morphogenesist/tissue development	Spliceosome
LOC_Os04g39610.1	Glycerophosphoryl diester phosphodiesterase family protein	−0.6	Shoot system development/cellular cell wall organization or biogenesis/ anatomical structure morphogenesis	Glycerophospholipid metabolism
LOC_Os05g35470.1	Dienelactone hydrolase family protein	−0.477	Shoot system development/shoot morphogenesis	Biosynthesis of secondary metabolites
LOC_Os10g02040.1	Peroxidase precursor	−0.456	Developmental growth involved in morphogenesis	Phenylalanine metabolism/ Phenylpropanoid biosynthesis
LOC_Os12g42980	Cysteine synthase	−0.384	Developmental process	Cysteine and methionine metabolism/ Sulfur metabolism
LOC_Os06g09679.1	Chaperonin	+1.736	Post-embryonic organ developmengan developmen/tissue development	
LOC_Os12g07650.1	OsGrx_S16 - glutaredoxin subgroup II	+2.027	Cellular cell wall organization or biogenesis/ anatomical structure morphogenesis/tissue development	
LOC_Os03g40830.1	OsSub30-Putative Subtilisin homologue	−0.581	Post-embryonic developmen/anatomical structure morphogenesis	
LOC_Os03g63480	Ankyrin repeat domain containing protein	+1.705	Post-embryonic development/cell differentiation/tissue development	

### Analysis of DGE libraries

**Figure 3:**
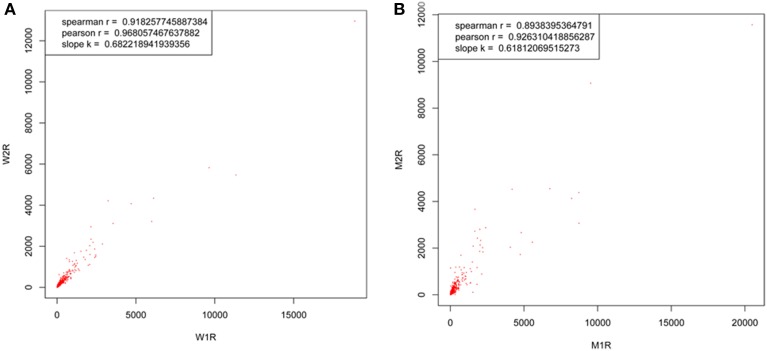
**Transcript correlation coefficients between two biological replicates of both wild-type samples (tags 116 and 117) and *dl2* mutant samples (tags 121 and 119)**. The ratios of quantified transcripts were plotted between two biological replicates of **(A)** wild type samples (W2R and W1R) and **(B)** mutant samples (M2R and M1R).

To compare the abundance changes between the proteome and transcriptome, we performed DGE experiments with mRNA samples from MT and WT samples. The correlation coefficient between two biological replicates in each experiment was evaluated first. As shown in Figure 3, both the correlation coefficients between the two biological replicates in the two experiments were approximately 0.9, and a much high overlap of the detected transcripts was exhibited in two biological replicates. This result demonstrated that the DGE libraries are applicable for further analysis. Approximately 3.4 million reads per sample were acquired on the Illumina GAIIx platform and above 96% of total reads were identified as clean reads, which were mapped to genes. Among the clean tags, 70–79% were mapped to the genes perfectly, and 12–17% were mapped to the genome (Table 3). To evaluate whether the sequencing depth was enough for transcriptome coverage, the sequencing saturation was examined in each example (Figure S1). The number of detected genes increased with the number of reads. However, when the number of reads reached more than 3 million, the rate of detected genes flattened, which indicated that the number of detected genes was saturated.

**Table 3:** **Summary of transcriptome of rice wild-type and *dl2* mutant**.

**Samples**	**Raw tags**	**Clean tags**	**Map to gene tags**	**Map to genome tags**	**Unknown tags**
M1R	3493296	3343333	2350431(70.30%)	481468(14.40%)	511434(15.30%)
M2R	3456666	3299843	2315846(70.18%)	540156(16.37%)	443841(13.45%)
W1R	3460174	3326656	2624430(78.89%)	403707(12.14%)	298519(8.97%)
W2R	3674583	3511076	2574523(73.33%)	486560(13.86%)	449993(12.82%)

### Verification of DEGs

**Figure 4:**
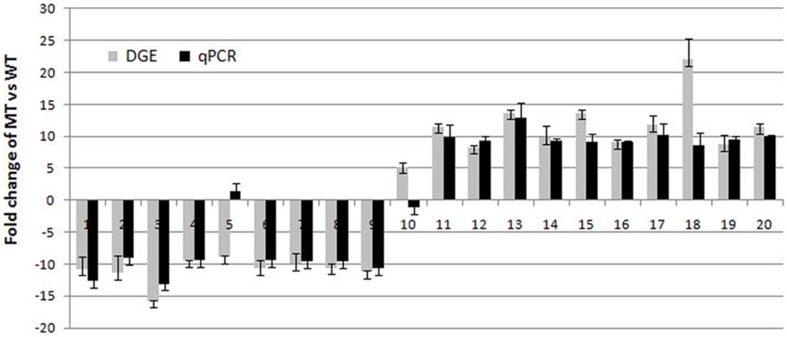
**Real-time quantitative PCR analyses of 20 genes selected randomly from the differentially expressed genes**. 1, LOC_Os01g10400.1; 2, LOC_Os02g18115.1; 3, LOC_Os12g24800.1; 4, LOC_Os05g33520.1; 5, LOC_Os10g10149.1; 6, LOC_Os03g25960.1; 7, LOC_Os11g35710.; 8, LOC_Os11g45750.1; 9, LOC_Os11g40140.1; 10, LOC_Os10g15164.1; 11, LOC_Os01g26039.1; 12, LOC_Os05g04520.1; 13, LOC_Os08g26840.1; 14, LOC_Os11g02670.1; 15, LOC_Os02g05000.1; 16, LOC_Os10g41650.1; 17, LOC_Os11g01074.2; 18, LOC_Os01g57260.1; 19, LOC_Os01g54400.1; 20, LOC_Os05g04530.1.

A total of 98 differentially expressed mRNAs (DEGs) showed a significant difference (*P* < 0.05) in the comparison, according to the more stringent criteria (ratio ≥ 2, *Q*-value ≥ 0.8) (Table S3). To validate the expression profiles obtained by RNA-Seq, qPCR was performed on 20 DEGs selected for increased or decreased expression levels (including transcription factors, protein-coding genes and unknown proteins) (Table S4). The absence of non-specific PCR products and primer dimers was confirmed by melting curve analysis and electrophoresis (data not shown). These selected genes encode proteins known to be related to, or involved in, plant tissue developmental processes in other species (Kleffmann et al., 2007; Cai et al., 2008; Vallabhaneni et al., 2010; Majeran et al., 2012). The transcript abundance patterns of MT and WT by qPCR analysis were compared with RNA-Seq data. The results showed that for 18 of the 20 genes, qPCR results displayed similar patterns as the RNA-Seq data, despite some quantitative differences in expression level (Figure 4). The remaining two genes showed different expression patterns by qPCR when compared to RNA-Seq. It is possible that the primers of these genes annealed within a non-specific or poorly annotated region.

### Comparison of cognate gene and protein expression

**Figure 5:**
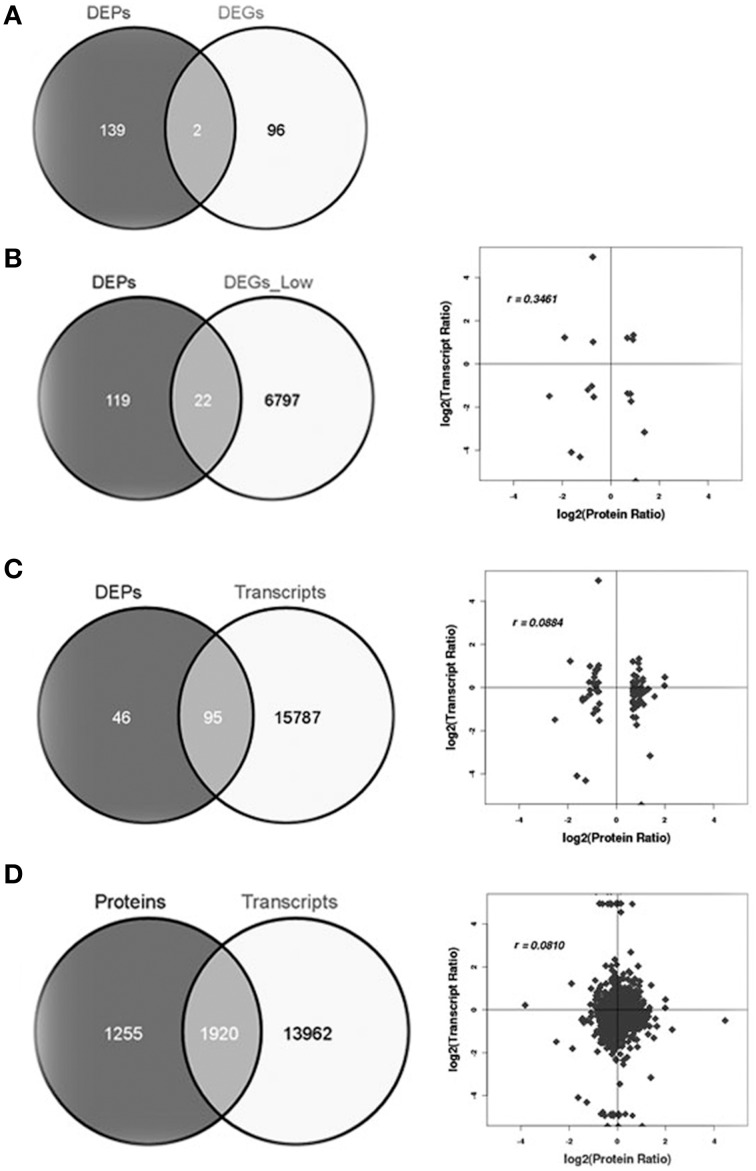
**Correlations between protein and transcript levels. (A)** Differentially expressed proteins and differentially expressed transcripts using stringent criteria, **(B)** differentially expressed proteins and differentially expressed transcripts with less stringent criteria, **(C)** differentially expressed proteins and all expressed transcripts, and **(D)** all expressed proteins and all expressed mRNA.

Integrative analysis of the proteome combined with a comprehensive transcriptome provides an important verification tool for the expression of key genes. To compare the proteome with the transcriptome, we compared the 141 DEPs with 98 differentially expressed mRNAs (DEGs). Surprisingly, only two genes overlapped according to the more stringent criteria (Figure 5A, Table S5). We then generated a second list correlating the 141 DEPs with the cognate mRNAs according to a less stringent criteria (ratio ≥ 2, no *Q*-value) (Table S6) and a positive correlation of *r* = 0.35 was obtained when all significantly changed proteins with a cognate mRNA were considered, despite differences in directions of regulation (Figure 5B). When DEPs were compared to all expressed mRNAs (Figure 5C and Table S7) a correlation of *r* = 0.088 was obtained. Finally, a correlation of *r* = 0.081 (Figure 5D) was obtained when all expressed protein was compared to all expressed mRNAs (Table S8). To reveal the correlation between DEPs and DEGs precisely, we calculated the correlation between the proteins with increased accumulation (Table S2) and mRNAs with increased accumulation (ratio ≥ 2, no *Q*-value) (Table S6), and a correlation of *r* = 0.3 is obtained (Figure S2A). Similarly, the correlation between proteins with decreased accumulation and mRNAs with decreased accumulation is *r* = 0.4 (Figure S2B). Generally, the relationship between mRNA and protein levels was modest between our analysis of the proteome and transcriptome.

Overall, proteins that exhibited notable changes in their expression levels did not have a corresponding alteration in transcript levels (Figure 5), indicating that post-transcription and post-translation may play important roles in regulating leaf vascular development. The correlation coefficient of the overall proteome and transcriptome data was very low (*r* = 0.081). This is expected because mRNA levels are not always consistent with protein levels due to post-transcriptional, translational and/or post-translational regulation (Gygi et al., 1999; Washburn et al., 2003). In our study, the average correlation between DEPs and DEGs is somewhat higher than that reported in other organisms, perhaps due to improvements in the LC-MS technology and/or statistical power.

## Discussion

### DEPs might be involved in leaf vascular development in rice

In this study, a modest correlation between the DEPs and DEGs was found. The up- or down-regulation of some key DEPs confirmed that the physiological process of vascular differentiation is complicated. The modest correlation between the DEPs and DEGs was most probably due to modulation of mRNA processing, selective mRNA translation, mRNA decay and/or protein synthesis and turnover (Galland et al., 2014). This behavior points out the importance of post-transcriptional regulatory mechanisms to account for the phenotype of the *dl2* rice mutant. We note in this context that proteins involved in spliceosome functioning were revealed in our study (Table 2, **Table S2**). mRNA processing, known as pre-mRNA splicing, is performed precisely and efficiently by the spliceosome. The spliceosome assembly and pre-mRNA splicing process requires a large set of proteins and the formation of protein–protein, protein–RNA, and RNA–RNA complexes (Jung and Kang, 2014). Post-transcriptional regulation, including mRNA processing, is emerging as a critical step in plant development. Therefore, alterations in spliceosome functioning could occur in *dl2* rice mutants compared to WT rice. To our knowledge, few mutations occurring in spliceosome proteins have been identified, essentially the *sad*, *sta1-1*, *Atsmu-1*, *Atsmu-2, smd3-a*, and *smd3-b* mutants of Arabidopsis (Xiong et al., 2001; Lee et al., 2006; Chung et al., 2009; Swaraz et al., 2011). As a component of the spliceosome, SART-1 protein accumulated in *dl2* mutants, compared to WT (Table 2). Consensus components for spliceosome structure suggest that the pre-mRNA splicing mechanism in plants could be similar to that in animals (Brown and Simpson, 1998; Lorkovic et al., 2000; Reddy, 2001; Swaraz et al., 2011), which could serve as a guide for directing genetic and functional analyses of spliceosome structure in plant.

It is noteworthy to mention that the protein methionine sulfoxide reductase MsrB (LOC_Os05g33510.1) showed an 8-fold increase in accumulation levels (Table S2), and its corresponding mRNA levels were also increased more than 8-fold in the *dl2* mutants compared to WT (Table S5). The Arabidopsis plastidic protein, MsrB, was shown to accumulate in response to a photooxidative treatment, which suggested a possible role of MsrB in tolerance to oxidative damage (Dos Santos et al., 2005). We also noted that both thioredoxin and glutaredoxin levels were increased significantly (Table S2). Thioredoxins and glutaredoxins constitute families of thiol oxidoreductases (Meyer et al., 2012), and it has been reported that the two oxidoreductases play a key role in protection against photooxidative stress in plants (Laporte et al., 2012; Wu et al., 2012; Rey et al., 2013). In our study, the result that the plastidic protein MsrB (LOC_Os05g33510.1), as well as thioredoxins (LOC_Os01g68480.1) and glutaredoxin (LOC_Os12g07650.1) were over-accumulated, suggests the occurrence of an oxidative stress in the *dl2* mutant, and the levels of these related enzymes were increased to repair defects in protein functions due to oxidation. Our previous results demonstrated that the *dl2 drooping leaf* phenotype is coupled with a defective response to auxin (Huang et al., 2011). Interestingly, the level of auxin-repressed protein (LOC_Os11g44810.1) was increased significantly (Table S2), which is consistent with the defective response to auxin in the *dl2* mutant. Recent research suggests that the plant growth regulator auxin may be the connecting link regulating the level of ROS and directing its role in oxidative damage or signaling in plants under stress (Bashandy et al., 2010; Krishnamurthy and Rathinasabapathi, 2013). Therefore, a likely hypothesis could be that this mutation entails an oxidative stress in rice *dl2* mutants. In the future, it will be interesting to investigate the participation of MsrB and related oxidoreductases during stress treatments by using knockout and overexpression mutants.

### Modest concordance between transcript and protein levels

To date, differential transcriptomic and proteomic experiments have provided an overview of the accumulation patterns of mRNAs and proteins (Chen et al., 2002; Fathi et al., 2009; Ghazalpour et al., 2011; Lan et al., 2011). However, the discordance between mRNA and protein abundance is present during the flow of genetic information from DNA to phenotype. In fact, protein levels are greatly influenced by translational and post-translational processing, and it was assumed that the selective mRNA translation and protein turnover contribute to the dynamic proteome (Galland et al., 2014). However, the extent to which this occurs is still poorly understood, and comprehending the correlation between the proteome and transcriptome has both practical and basic implications. As we have known, genetic studies regard transcription alterations as a function of genetic variation and employ the data to make models, such as interaction networks, to explain complex phenotypes (Brem et al., 2002; Schadt et al., 2003; Keurentjes et al., 2007). Systems based on these methods, in particular, have relied heavily on transcriptome data. However, there are widely reported deviations between the proteome and transcriptome (Ghazalpour et al., 2011; Galland et al., 2014), which has been further highlighted by our study.

We have previously reported on the *dl2* mutantin rice with midribless leaf and altered plant shape (Huang et al., 2011). Here, a comparative analysis of the genetic regulation of transcriptome and proteome in *dl2* mutants was performed. By checking the effects of thousands of genetic disturbance simultaneously at the transcript and protein level, we were able to evaluate the global nature of relationships between the two levels objectively. This analysis showed that the relationship between the protein and transcript expression was modest. We predicted that the heredity variation is a predictor of concordance between protein and transcript levels. Our results complement the data previously reported for yeast and plant, indicating similar modest protein-transcript correlation. Compared to yeast (Foss et al., 2007), our results showed a much lower estimate of protein-transcript concordance (0.081 vs. 0.18 correlation) when considering all the protein measurements, and a significantly higher estimate (0.34 vs. 0.18 correlation) when considering DEPs. The slightly higher correlation between the proteome and transcriptome in our study is probably due to advancements in the LC-MS technology and/or analysis tools. The strategy of iTRAQ, which provides the advantage of overcoming peptide level alterations due to platform improvements, is known to be more precise in quantifying peptides when compared to label free methods (Ghazalpour et al., 2011). Overall, we found that the relationship between differentially expressed protein and differentially expressed mRNA was modest (under *r* = 0.34), and the DEPs may be considered as candidates involved in the physiological process of leaf vascular pattern, but it requires further study.

### Conflict of interest statement

The authors declare that the research was conducted in the absence of any commercial or financial relationships that could be construed as a potential conflict of interest.

## Supplementary material

Figure S1 **Analysis of sequence saturation for four samples from wild type (WT) and mutant MT**. Samples W1R and W2R were from WT and M1R and M2R were from MT.

Figure S2 **Correlations between protein and transcript levels. (A)** Correlation between differentially expressed proteins (DEPs) with increased accumulation and differentially expressed genes (DEGs) with increased accumulation, **(B)** Correlation between DEPs with decreased accumulation and DEGs with decreased accumulation.

Table S1 **A total of 3172 proteins in two experiments were identified with higher significance (*P* < 0.05)**.

Table S2 **141 DEPs (differentially expressed proteins) (*P* < 0.05, and the fold change ≥1.6)**.

Table S3 **A total of 98 DEGs (differentially expressed genes) (*p*-values < 0.05, and the fold change ≥ 2.0, *Q* ≥ 0.8)**.

Table S4 **Primers for real-time quantitative PCR analysis**.

Table S5 **Correlation analysis between differentially expressed proteins (DEPs) and differentially expressed genes (DEGs) with more stringent criteria (Ratio ≥ 2, *Q*-value ≥ 0.8)**.

Table S6 **Correlation analysis between differentially expressed proteins (DEPs) and differentially expressed genes (DEGs) with less stringent criteria (Ratio ≥ 2, no *Q*-value)**.

Table S7 **Correlation analysis between differentially expressed proteins (DEPs) and all expressed genes (DEGs)**.

Table S8 **Correlation analysis between all expressed proteins (DEPs) and all expressed genes (DEGs)**.

## Abbreviations

iTRAQ: isobaric tag for relative and absolute quantification
DGE: digital gene expression
DEGs: differentially expressed genes
DEPs: differentially expressed proteins
MT: mutant
WT: wild type.
