# Supplementary figures and images for: Integration of the proteome and transcriptome reveals multiple levels of gene regulation in the rice dl2 mutant
Source: Front Plant Sci. 2015 Jun 17;6:351. doi: 10.3389/fpls.2015.00351 (PMC4469824; doi:10.3389/fpls.2015.00351)

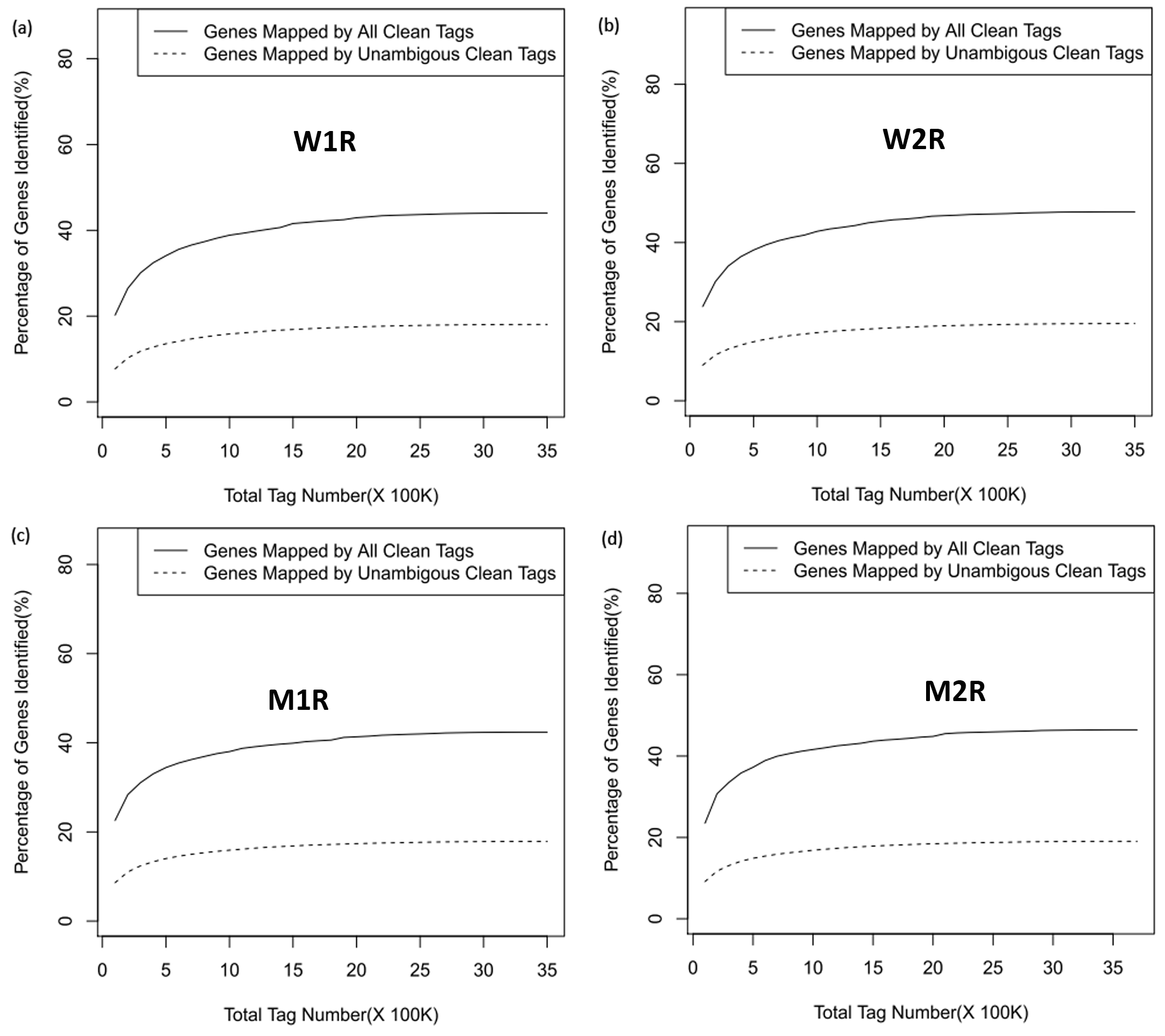

Supplement: Figure S1 — Analysis of sequence saturation for four samples from wild type (WT) and mutant MT. Samples W1R and W2R were from WT and M1R and M2R were from MT. [file Image1.TIF]

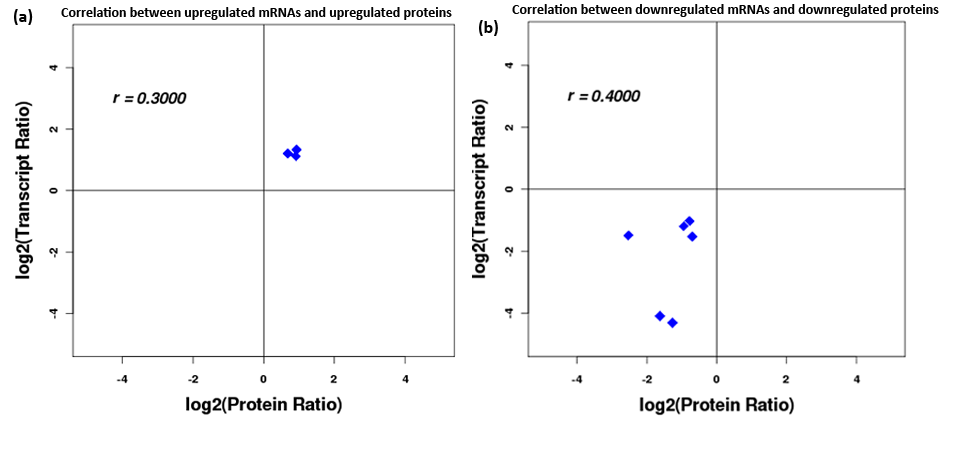

Supplement: Figure S2 — Correlations between protein and transcript levels. (A) Correlation between differentially expressed proteins (DEPs) with increased accumulation and differentially expressed genes (DEGs) with increased accumulation, (B) Correlation between DEPs with decreased accumulation and DEGs with decreased accumulation. [file Image2.TIF]
