# Supplementary material for: Integration of the proteome and transcriptome reveals multiple levels of gene regulation in the rice dl2 mutant
Source: Front Plant Sci. 2015 Jun 17;6:351. doi: 10.3389/fpls.2015.00351 (PMC4469824; doi:10.3389/fpls.2015.00351)
Supplement: Table S4 — Primers for real-time quantitative PCR analysis. [file Table4.DOC]

Table 4 Primers for qPCR

| Number | Gene ID | Primers(5’ to 3’) |
| --- | --- | --- |
| 1 | LOC_Os01g10400.1 | FW: CAAGCCTCGCCTTCCAAA  RV: TCCTCGTAGCCGACCTCCT |
| 2 | LOC_Os02g18115.1 | FW: TCATTCCGCTCGCTCGTC  RV: TTCCAAAGCCAGCCCAAAC |
| 3 | LOC_Os12g24800.1 | FW: CACTCCCTTCTCATTCCC  RV: AGCCCTTGTTCAGGTTAA |
| 4 | LOC_Os05g33520.1 | AGTTCAGAAGACCGCAGCAA  GGACCACATTCCCATCAGC |
| 5 | LOC_Os10g10149.1 | FW: TGTGCGACCCTCAGTAAGC  RV: GTAGAGTGGAAGATGGCGAAG |
| 6 | LOC_Os03g25960.1 | FW: AAGGGAGCAATCATCGCAGAG  RV: AGCGGAACCATACGTGACAAAA |
| 7 | LOC_Os11g35710.1 | FW: TATGTCACCCTGAGACTTATC  RV: ACGAACTTCTTGCCGTAC |
| 8 | LOC_Os11g45750.1 | FW: TCGCCGTAAGTTCAATCA  RV: CTCCTCCAGTAGTAGAAGCAAT |
| 9 | LOC_Os11g40140.1 | FW: ACGACCATTTGGTGTCTC  RV: TTCTTCCATAACCTGCTTC |
| 10 | LOC_Os10g15164.1 | FW: CGATGAACGCTGACACTA  RV: GAAGCAGGAAACAAGAAAT |
| 11 | LOC_Os01g26039.1 | FW: AGCAGCCTACGCTAACTC  RV: TAACACCGTCCTCCAACA |
| 12 | LOC_Os05g04520.1 | FW: GGCGTTATGCGTTCAGTA  RV: TTCTCCAGGGTTAGTTGC |
| 13 | LOC_Os08g26840.1 | FW: GAACTCGCAAGCCTGAACTCG  RV:GGAGCCTACCCACCTTGTCG |
| 14 | LOC_Os11g02670.1 | FW: TATGAAAGGTCCAAGCAA  RV: CAGCCATCTGACGAGTTA |
| 15 | LOC_Os02g05000.1 | FW: CGGTTTGTTTATCTGGTGTT  RV: ACTTGGAGAATGGAGGGTAG |
| 16 | LOC_Os10g41650.1 | FW: CTCGGCTGCCCGTCAAAT  RV: TATCGCAACGCCCAAGGA |
| 17 | LOC_Os11g01074.2 | FW: TCTCACCGAAGCATCAAG  RV: GCTGGCAAGGTAGACAATA |
| 18 | LOC_Os01g57260.1 | FW: TGCGTCCAGAGCTTTATTACCG  RV: TCACCTTGACCTTCCCTTCG |
| 19 | LOC_Os01g54400.1 | FW: CCGTCGTCTTCCACCTCGTC  RV: GCGGCGTTCTTGGAGGGTT |
| 20 | LOC_Os05g04530.1 | FW: TCTGCCTTCAGGAATCAC  RV: GTATGGGACACTGGGTTG |
